# Supplementary figures and images for: An effective transformer based on dual attention fusion for underwater image enhancement (part 2 of 2)
Source: PeerJ Comput Sci. 2024 Apr 30;10:e1783. doi: 10.7717/peerj-cs.1783 (PMC11157557; doi:10.7717/peerj-cs.1783)

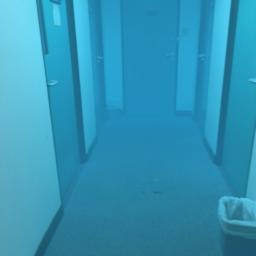

Supplement: Supplemental Information 2 — Experimental results of images on the test set. [file peerj-cs-10-1783-s002.zip › distorted/kinect2data_2598_typeC.jpg]

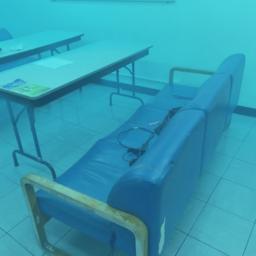

Supplement: Supplemental Information 2 — Experimental results of images on the test set. [file peerj-cs-10-1783-s002.zip › distorted/kinect2data_2621_typeC.jpg]

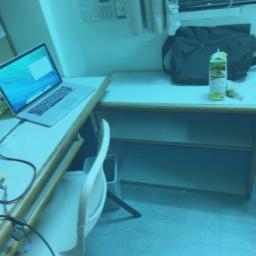

Supplement: Supplemental Information 2 — Experimental results of images on the test set. [file peerj-cs-10-1783-s002.zip › distorted/kinect2data_2704_typeC.jpg]

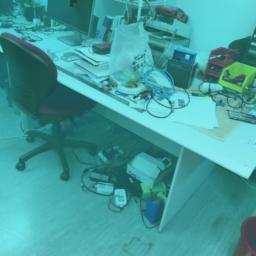

Supplement: Supplemental Information 2 — Experimental results of images on the test set. [file peerj-cs-10-1783-s002.zip › distorted/kinect2data_2717_typeC.jpg]

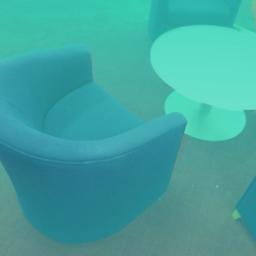

Supplement: Supplemental Information 2 — Experimental results of images on the test set. [file peerj-cs-10-1783-s002.zip › distorted/kinect2data_2727_typeD.jpg]

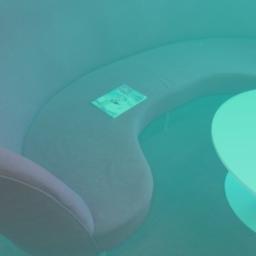

Supplement: Supplemental Information 2 — Experimental results of images on the test set. [file peerj-cs-10-1783-s002.zip › distorted/kinect2data_2732_typeD.jpg]

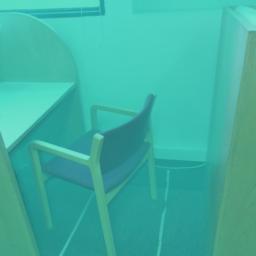

Supplement: Supplemental Information 2 — Experimental results of images on the test set. [file peerj-cs-10-1783-s002.zip › distorted/kinect2data_2735_typeD.jpg]

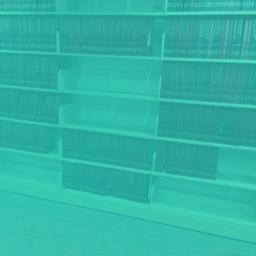

Supplement: Supplemental Information 2 — Experimental results of images on the test set. [file peerj-cs-10-1783-s002.zip › distorted/kinect2data_2744_typeD.jpg]

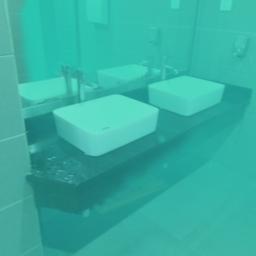

Supplement: Supplemental Information 2 — Experimental results of images on the test set. [file peerj-cs-10-1783-s002.zip › distorted/kinect2data_2785_typeD.jpg]

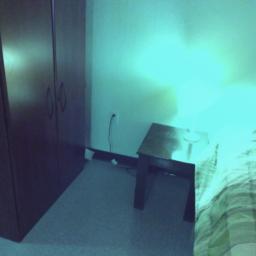

Supplement: Supplemental Information 2 — Experimental results of images on the test set. [file peerj-cs-10-1783-s002.zip › distorted/kinect2data_279_typeB.jpg]

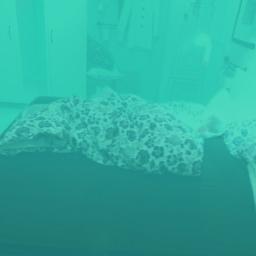

Supplement: Supplemental Information 2 — Experimental results of images on the test set. [file peerj-cs-10-1783-s002.zip › distorted/kinect2data_2824_typeD.jpg]

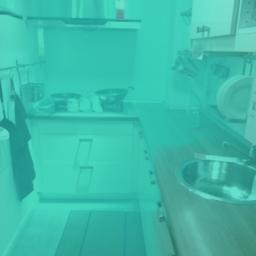

Supplement: Supplemental Information 2 — Experimental results of images on the test set. [file peerj-cs-10-1783-s002.zip › distorted/kinect2data_2849_typeD.jpg]

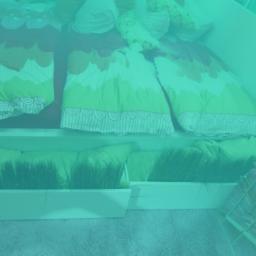

Supplement: Supplemental Information 2 — Experimental results of images on the test set. [file peerj-cs-10-1783-s002.zip › distorted/kinect2data_2879_typeD.jpg]

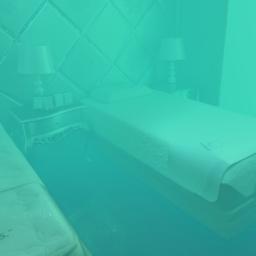

Supplement: Supplemental Information 2 — Experimental results of images on the test set. [file peerj-cs-10-1783-s002.zip › distorted/kinect2data_2907_typeD.jpg]

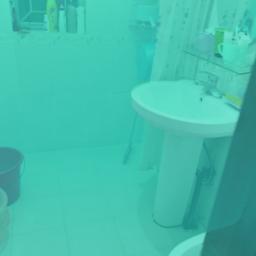

Supplement: Supplemental Information 2 — Experimental results of images on the test set. [file peerj-cs-10-1783-s002.zip › distorted/kinect2data_2933_typeD.jpg]

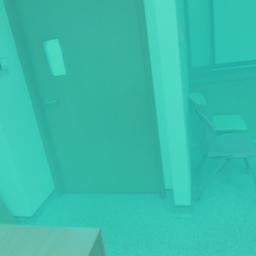

Supplement: Supplemental Information 2 — Experimental results of images on the test set. [file peerj-cs-10-1783-s002.zip › distorted/kinect2data_2951_typeD.jpg]

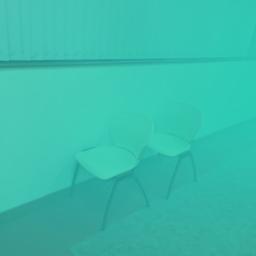

Supplement: Supplemental Information 2 — Experimental results of images on the test set. [file peerj-cs-10-1783-s002.zip › distorted/kinect2data_2973_typeD.jpg]

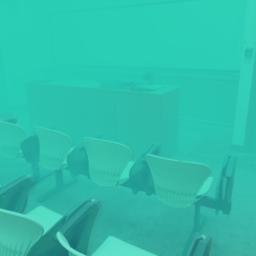

Supplement: Supplemental Information 2 — Experimental results of images on the test set. [file peerj-cs-10-1783-s002.zip › distorted/kinect2data_2977_typeD.jpg]

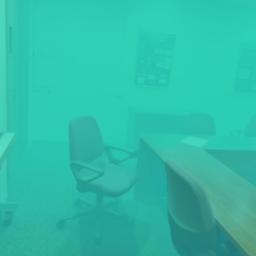

Supplement: Supplemental Information 2 — Experimental results of images on the test set. [file peerj-cs-10-1783-s002.zip › distorted/kinect2data_2989_typeD.jpg]

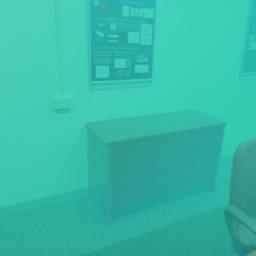

Supplement: Supplemental Information 2 — Experimental results of images on the test set. [file peerj-cs-10-1783-s002.zip › distorted/kinect2data_2990_typeD.jpg]

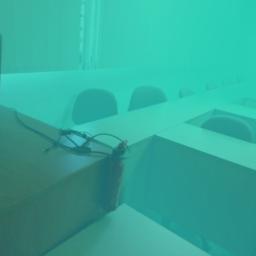

Supplement: Supplemental Information 2 — Experimental results of images on the test set. [file peerj-cs-10-1783-s002.zip › distorted/kinect2data_3068_typeD.jpg]

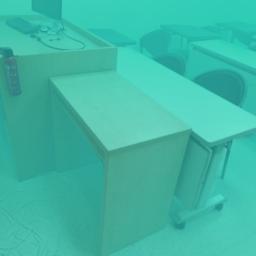

Supplement: Supplemental Information 2 — Experimental results of images on the test set. [file peerj-cs-10-1783-s002.zip › distorted/kinect2data_3078_typeD.jpg]

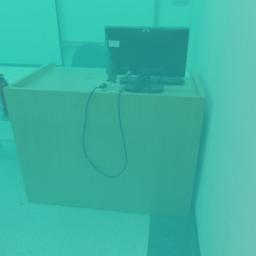

Supplement: Supplemental Information 2 — Experimental results of images on the test set. [file peerj-cs-10-1783-s002.zip › distorted/kinect2data_3091_typeD.jpg]

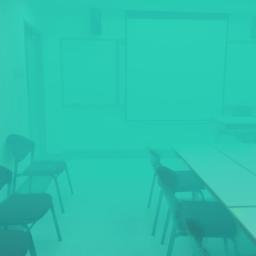

Supplement: Supplemental Information 2 — Experimental results of images on the test set. [file peerj-cs-10-1783-s002.zip › distorted/kinect2data_3095_typeD.jpg]

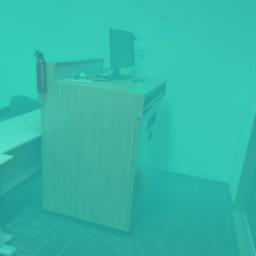

Supplement: Supplemental Information 2 — Experimental results of images on the test set. [file peerj-cs-10-1783-s002.zip › distorted/kinect2data_3097_typeD.jpg]

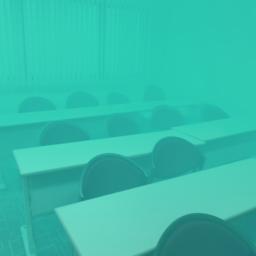

Supplement: Supplemental Information 2 — Experimental results of images on the test set. [file peerj-cs-10-1783-s002.zip › distorted/kinect2data_3104_typeD.jpg]

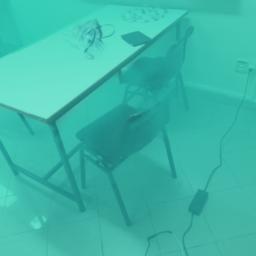

Supplement: Supplemental Information 2 — Experimental results of images on the test set. [file peerj-cs-10-1783-s002.zip › distorted/kinect2data_3121_typeD.jpg]

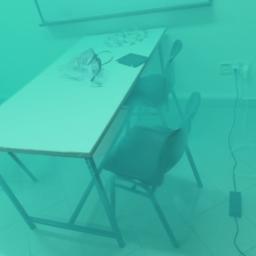

Supplement: Supplemental Information 2 — Experimental results of images on the test set. [file peerj-cs-10-1783-s002.zip › distorted/kinect2data_3124_typeD.jpg]

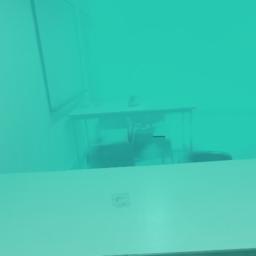

Supplement: Supplemental Information 2 — Experimental results of images on the test set. [file peerj-cs-10-1783-s002.zip › distorted/kinect2data_3135_typeD.jpg]

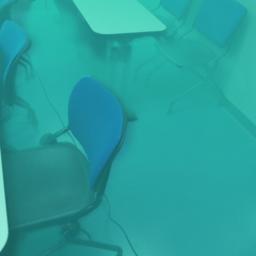

Supplement: Supplemental Information 2 — Experimental results of images on the test set. [file peerj-cs-10-1783-s002.zip › distorted/kinect2data_3166_typeD.jpg]

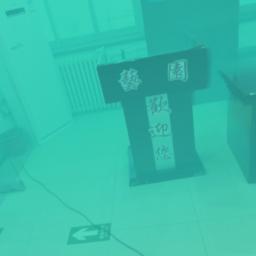

Supplement: Supplemental Information 2 — Experimental results of images on the test set. [file peerj-cs-10-1783-s002.zip › distorted/kinect2data_3183_typeD.jpg]

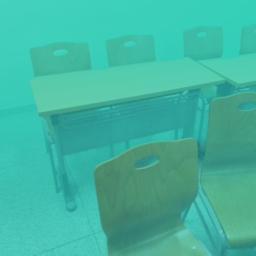

Supplement: Supplemental Information 2 — Experimental results of images on the test set. [file peerj-cs-10-1783-s002.zip › distorted/kinect2data_3215_typeD.jpg]

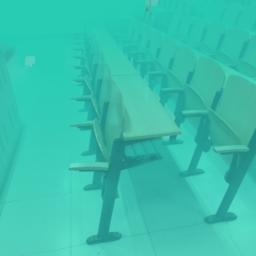

Supplement: Supplemental Information 2 — Experimental results of images on the test set. [file peerj-cs-10-1783-s002.zip › distorted/kinect2data_3219_typeD.jpg]

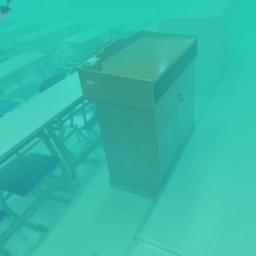

Supplement: Supplemental Information 2 — Experimental results of images on the test set. [file peerj-cs-10-1783-s002.zip › distorted/kinect2data_3228_typeD.jpg]

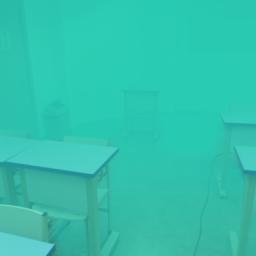

Supplement: Supplemental Information 2 — Experimental results of images on the test set. [file peerj-cs-10-1783-s002.zip › distorted/kinect2data_3233_typeD.jpg]

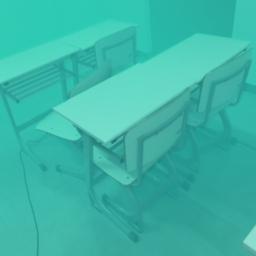

Supplement: Supplemental Information 2 — Experimental results of images on the test set. [file peerj-cs-10-1783-s002.zip › distorted/kinect2data_3259_typeD.jpg]

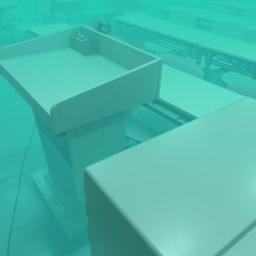

Supplement: Supplemental Information 2 — Experimental results of images on the test set. [file peerj-cs-10-1783-s002.zip › distorted/kinect2data_3266_typeD.jpg]

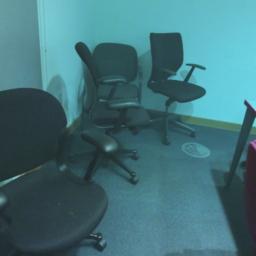

Supplement: Supplemental Information 2 — Experimental results of images on the test set. [file peerj-cs-10-1783-s002.zip › distorted/kinect2data_326_typeB.jpg]

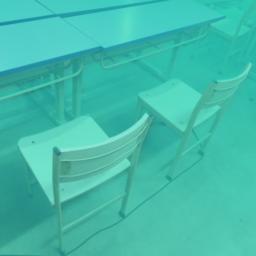

Supplement: Supplemental Information 2 — Experimental results of images on the test set. [file peerj-cs-10-1783-s002.zip › distorted/kinect2data_3292_typeD.jpg]

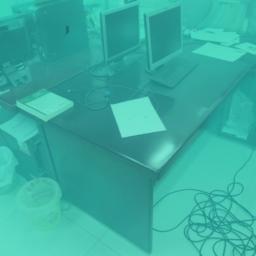

Supplement: Supplemental Information 2 — Experimental results of images on the test set. [file peerj-cs-10-1783-s002.zip › distorted/kinect2data_3311_typeD.jpg]

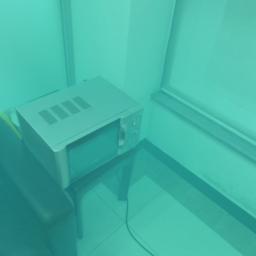

Supplement: Supplemental Information 2 — Experimental results of images on the test set. [file peerj-cs-10-1783-s002.zip › distorted/kinect2data_3318_typeD.jpg]

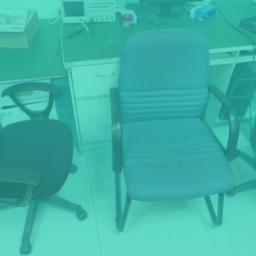

Supplement: Supplemental Information 2 — Experimental results of images on the test set. [file peerj-cs-10-1783-s002.zip › distorted/kinect2data_3326_typeD.jpg]

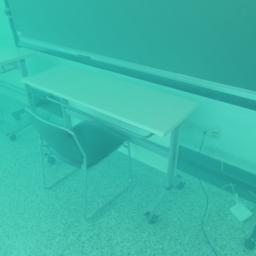

Supplement: Supplemental Information 2 — Experimental results of images on the test set. [file peerj-cs-10-1783-s002.zip › distorted/kinect2data_3329_typeD.jpg]

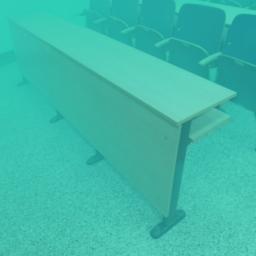

Supplement: Supplemental Information 2 — Experimental results of images on the test set. [file peerj-cs-10-1783-s002.zip › distorted/kinect2data_3339_typeD.jpg]

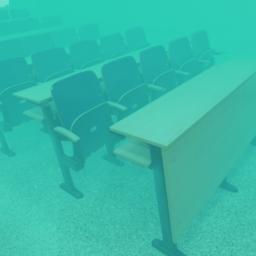

Supplement: Supplemental Information 2 — Experimental results of images on the test set. [file peerj-cs-10-1783-s002.zip › distorted/kinect2data_3345_typeD.jpg]

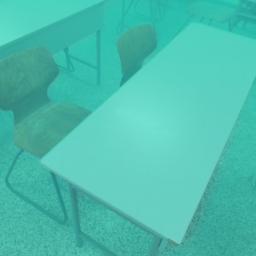

Supplement: Supplemental Information 2 — Experimental results of images on the test set. [file peerj-cs-10-1783-s002.zip › distorted/kinect2data_3357_typeD.jpg]

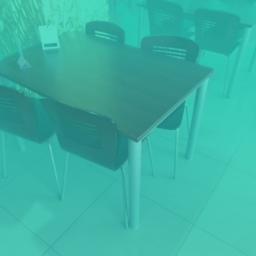

Supplement: Supplemental Information 2 — Experimental results of images on the test set. [file peerj-cs-10-1783-s002.zip › distorted/kinect2data_3375_typeD.jpg]

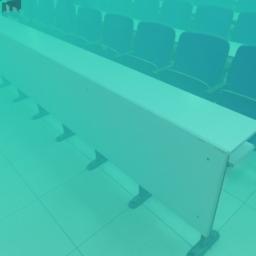

Supplement: Supplemental Information 2 — Experimental results of images on the test set. [file peerj-cs-10-1783-s002.zip › distorted/kinect2data_3387_typeD.jpg]

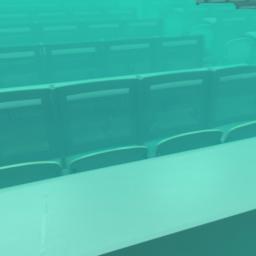

Supplement: Supplemental Information 2 — Experimental results of images on the test set. [file peerj-cs-10-1783-s002.zip › distorted/kinect2data_3415_typeD.jpg]

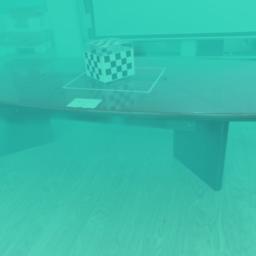

Supplement: Supplemental Information 2 — Experimental results of images on the test set. [file peerj-cs-10-1783-s002.zip › distorted/kinect2data_3440_typeD.jpg]

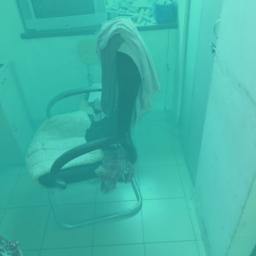

Supplement: Supplemental Information 2 — Experimental results of images on the test set. [file peerj-cs-10-1783-s002.zip › distorted/kinect2data_3453_typeD.jpg]

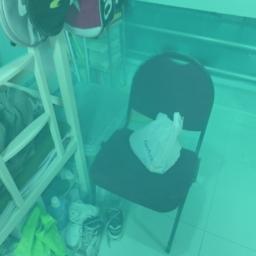

Supplement: Supplemental Information 2 — Experimental results of images on the test set. [file peerj-cs-10-1783-s002.zip › distorted/kinect2data_3475_typeD.jpg]

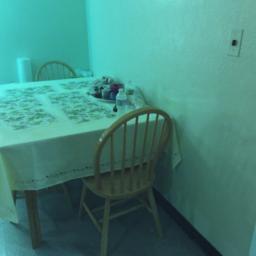

Supplement: Supplemental Information 2 — Experimental results of images on the test set. [file peerj-cs-10-1783-s002.zip › distorted/kinect2data_348_typeB.jpg]

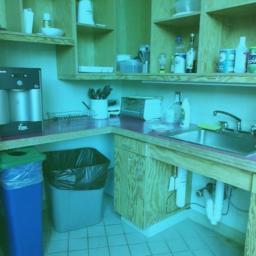

Supplement: Supplemental Information 2 — Experimental results of images on the test set. [file peerj-cs-10-1783-s002.zip › distorted/kinect2data_363_typeB.jpg]

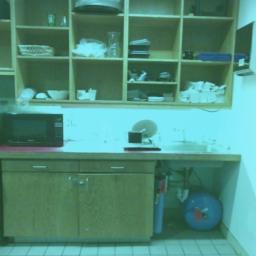

Supplement: Supplemental Information 2 — Experimental results of images on the test set. [file peerj-cs-10-1783-s002.zip › distorted/kinect2data_372_typeB.jpg]

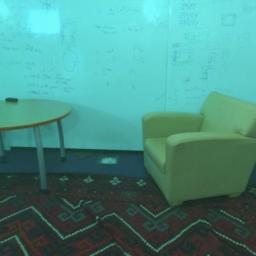

Supplement: Supplemental Information 2 — Experimental results of images on the test set. [file peerj-cs-10-1783-s002.zip › distorted/kinect2data_475_typeB.jpg]

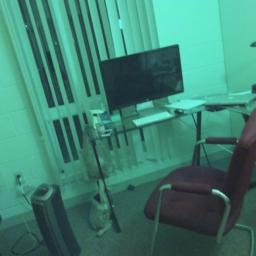

Supplement: Supplemental Information 2 — Experimental results of images on the test set. [file peerj-cs-10-1783-s002.zip › distorted/kinect2data_48_typeB.jpg]

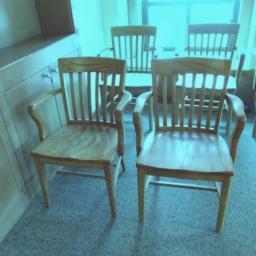

Supplement: Supplemental Information 2 — Experimental results of images on the test set. [file peerj-cs-10-1783-s002.zip › distorted/kinect2data_50_typeB.jpg]

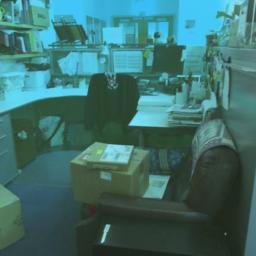

Supplement: Supplemental Information 2 — Experimental results of images on the test set. [file peerj-cs-10-1783-s002.zip › distorted/kinect2data_518_typeB.jpg]

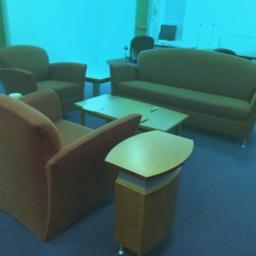

Supplement: Supplemental Information 2 — Experimental results of images on the test set. [file peerj-cs-10-1783-s002.zip › distorted/kinect2data_577_typeB.jpg]

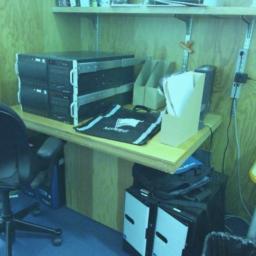

Supplement: Supplemental Information 2 — Experimental results of images on the test set. [file peerj-cs-10-1783-s002.zip › distorted/kinect2data_623_typeB.jpg]

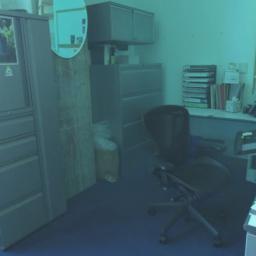

Supplement: Supplemental Information 2 — Experimental results of images on the test set. [file peerj-cs-10-1783-s002.zip › distorted/kinect2data_658_typeB.jpg]

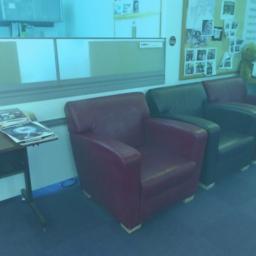

Supplement: Supplemental Information 2 — Experimental results of images on the test set. [file peerj-cs-10-1783-s002.zip › distorted/kinect2data_659_typeB.jpg]

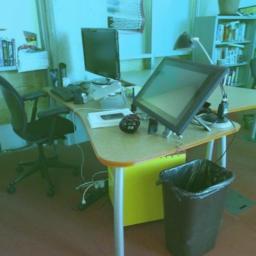

Supplement: Supplemental Information 2 — Experimental results of images on the test set. [file peerj-cs-10-1783-s002.zip › distorted/kinect2data_675_typeB.jpg]

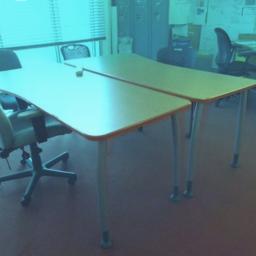

Supplement: Supplemental Information 2 — Experimental results of images on the test set. [file peerj-cs-10-1783-s002.zip › distorted/kinect2data_677_typeB.jpg]

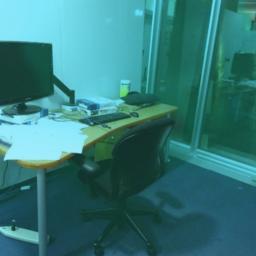

Supplement: Supplemental Information 2 — Experimental results of images on the test set. [file peerj-cs-10-1783-s002.zip › distorted/kinect2data_732_typeB.jpg]

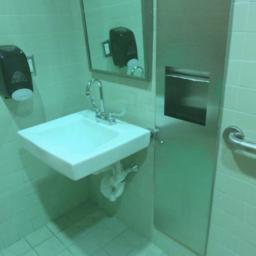

Supplement: Supplemental Information 2 — Experimental results of images on the test set. [file peerj-cs-10-1783-s002.zip › distorted/kinect2data_746_typeB.jpg]

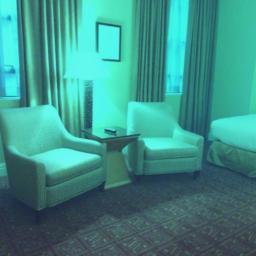

Supplement: Supplemental Information 2 — Experimental results of images on the test set. [file peerj-cs-10-1783-s002.zip › distorted/kinect2data_763_typeB.jpg]

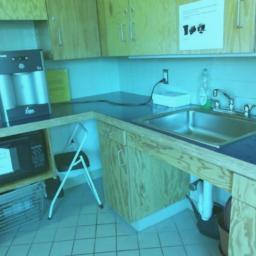

Supplement: Supplemental Information 2 — Experimental results of images on the test set. [file peerj-cs-10-1783-s002.zip › distorted/kinect2data_773_typeB.jpg]

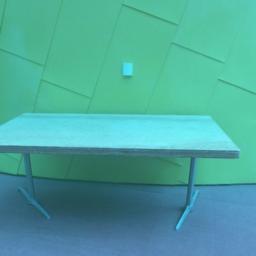

Supplement: Supplemental Information 2 — Experimental results of images on the test set. [file peerj-cs-10-1783-s002.zip › distorted/kinect2data_832_typeB.jpg]

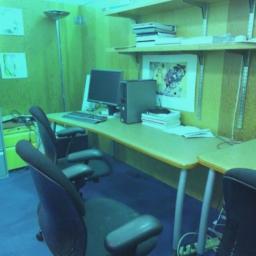

Supplement: Supplemental Information 2 — Experimental results of images on the test set. [file peerj-cs-10-1783-s002.zip › distorted/kinect2data_943_typeB.jpg]

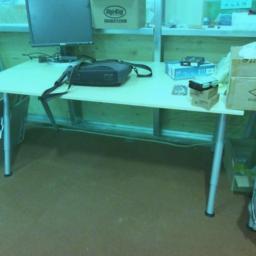

Supplement: Supplemental Information 2 — Experimental results of images on the test set. [file peerj-cs-10-1783-s002.zip › distorted/kinect2data_948_typeB.jpg]

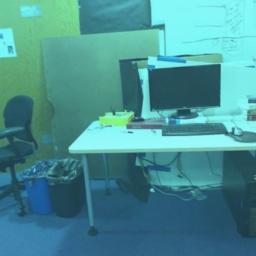

Supplement: Supplemental Information 2 — Experimental results of images on the test set. [file peerj-cs-10-1783-s002.zip › distorted/kinect2data_969_typeB.jpg]

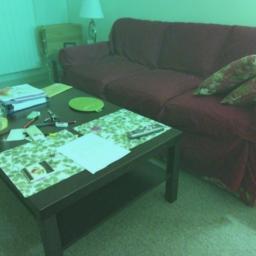

Supplement: Supplemental Information 2 — Experimental results of images on the test set. [file peerj-cs-10-1783-s002.zip › distorted/kinect2data_96_typeB.jpg]

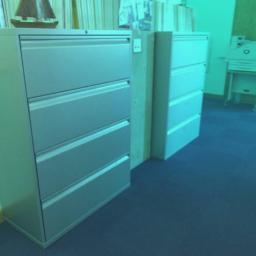

Supplement: Supplemental Information 2 — Experimental results of images on the test set. [file peerj-cs-10-1783-s002.zip › distorted/kinect2data_977_typeB.jpg]

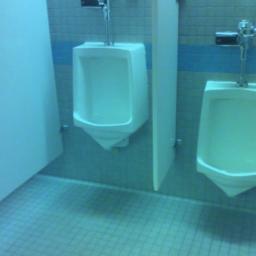

Supplement: Supplemental Information 2 — Experimental results of images on the test set. [file peerj-cs-10-1783-s002.zip › distorted/lg_104_typeB.jpg]

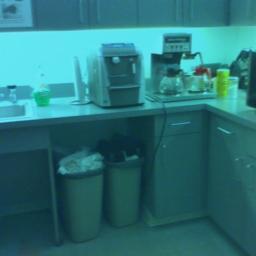

Supplement: Supplemental Information 2 — Experimental results of images on the test set. [file peerj-cs-10-1783-s002.zip › distorted/lg_164_typeB.jpg]

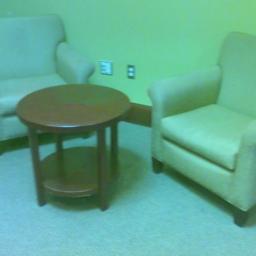

Supplement: Supplemental Information 2 — Experimental results of images on the test set. [file peerj-cs-10-1783-s002.zip › distorted/lg_217_typeB.jpg]

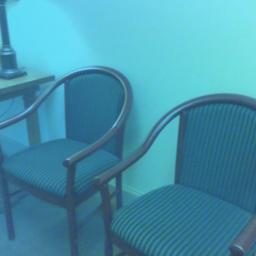

Supplement: Supplemental Information 2 — Experimental results of images on the test set. [file peerj-cs-10-1783-s002.zip › distorted/lg_300_typeC.jpg]

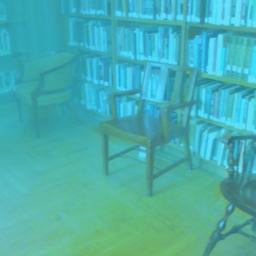

Supplement: Supplemental Information 2 — Experimental results of images on the test set. [file peerj-cs-10-1783-s002.zip › distorted/lg_325_typeC.jpg]

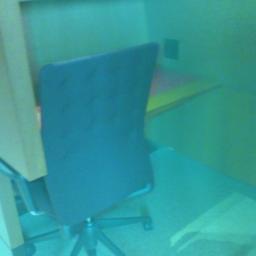

Supplement: Supplemental Information 2 — Experimental results of images on the test set. [file peerj-cs-10-1783-s002.zip › distorted/lg_349_typeC.jpg]

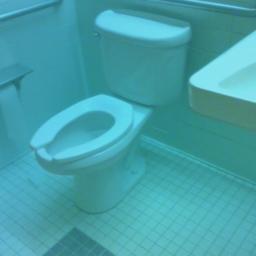

Supplement: Supplemental Information 2 — Experimental results of images on the test set. [file peerj-cs-10-1783-s002.zip › distorted/lg_389_typeC.jpg]

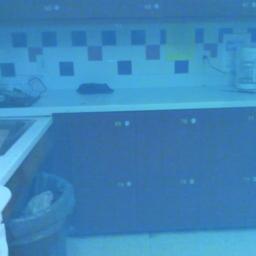

Supplement: Supplemental Information 2 — Experimental results of images on the test set. [file peerj-cs-10-1783-s002.zip › distorted/lg_449_typeC.jpg]

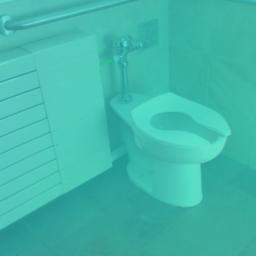

Supplement: Supplemental Information 2 — Experimental results of images on the test set. [file peerj-cs-10-1783-s002.zip › distorted/lg_506_typeD.jpg]

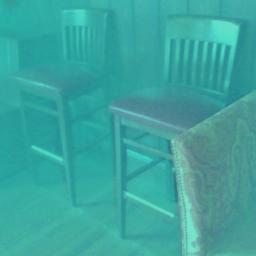

Supplement: Supplemental Information 2 — Experimental results of images on the test set. [file peerj-cs-10-1783-s002.zip › distorted/lg_513_typeD.jpg]

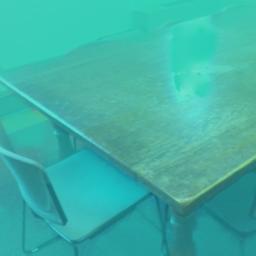

Supplement: Supplemental Information 2 — Experimental results of images on the test set. [file peerj-cs-10-1783-s002.zip › distorted/lg_531_typeD.jpg]

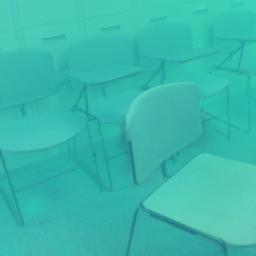

Supplement: Supplemental Information 2 — Experimental results of images on the test set. [file peerj-cs-10-1783-s002.zip › distorted/lg_534_typeD.jpg]

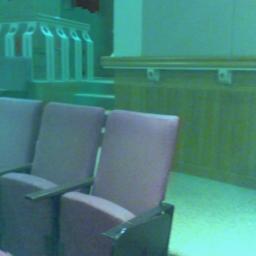

Supplement: Supplemental Information 2 — Experimental results of images on the test set. [file peerj-cs-10-1783-s002.zip › distorted/lg_95_typeB.jpg]

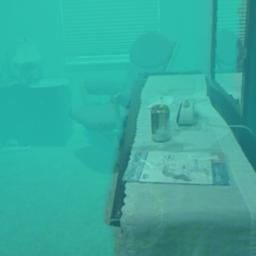

Supplement: Supplemental Information 2 — Experimental results of images on the test set. [file peerj-cs-10-1783-s002.zip › distorted/NYUdata_1047_typeD.jpg]

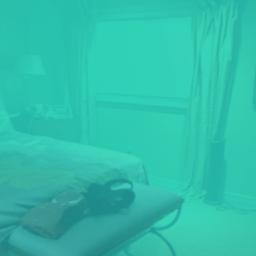

Supplement: Supplemental Information 2 — Experimental results of images on the test set. [file peerj-cs-10-1783-s002.zip › distorted/NYUdata_1074_typeD.jpg]

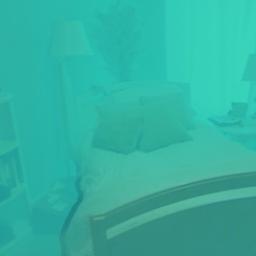

Supplement: Supplemental Information 2 — Experimental results of images on the test set. [file peerj-cs-10-1783-s002.zip › distorted/NYUdata_1091_typeD.jpg]

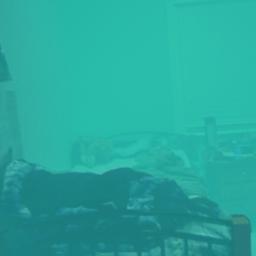

Supplement: Supplemental Information 2 — Experimental results of images on the test set. [file peerj-cs-10-1783-s002.zip › distorted/NYUdata_1122_typeD.jpg]

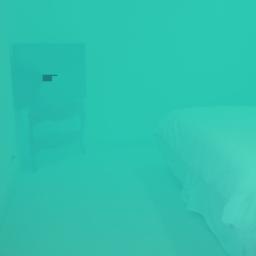

Supplement: Supplemental Information 2 — Experimental results of images on the test set. [file peerj-cs-10-1783-s002.zip › distorted/NYUdata_1137_typeD.jpg]

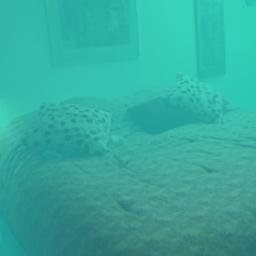

Supplement: Supplemental Information 2 — Experimental results of images on the test set. [file peerj-cs-10-1783-s002.zip › distorted/NYUdata_1173_typeD.jpg]

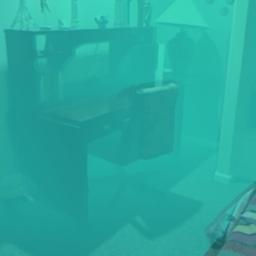

Supplement: Supplemental Information 2 — Experimental results of images on the test set. [file peerj-cs-10-1783-s002.zip › distorted/NYUdata_1179_typeD.jpg]

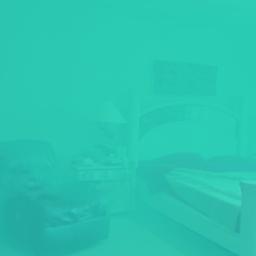

Supplement: Supplemental Information 2 — Experimental results of images on the test set. [file peerj-cs-10-1783-s002.zip › distorted/NYUdata_1186_typeD.jpg]

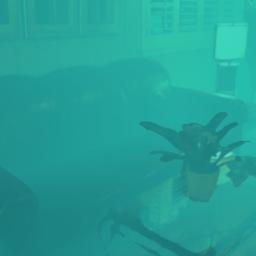

Supplement: Supplemental Information 2 — Experimental results of images on the test set. [file peerj-cs-10-1783-s002.zip › distorted/NYUdata_1230_typeD.jpg]

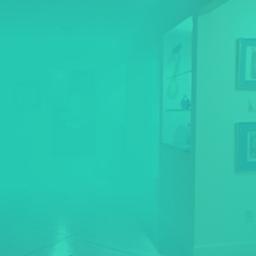

Supplement: Supplemental Information 2 — Experimental results of images on the test set. [file peerj-cs-10-1783-s002.zip › distorted/NYUdata_1255_typeD.jpg]

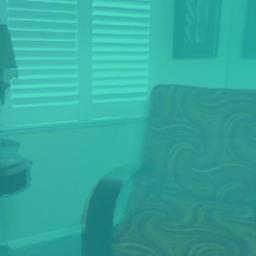

Supplement: Supplemental Information 2 — Experimental results of images on the test set. [file peerj-cs-10-1783-s002.zip › distorted/NYUdata_1268_typeD.jpg]

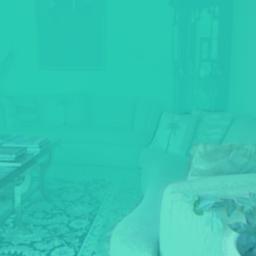

Supplement: Supplemental Information 2 — Experimental results of images on the test set. [file peerj-cs-10-1783-s002.zip › distorted/NYUdata_1270_typeD.jpg]
