# Supplementary figures and images for: An effective transformer based on dual attention fusion for underwater image enhancement (part 1 of 2)
Source: PeerJ Comput Sci. 2024 Apr 30;10:e1783. doi: 10.7717/peerj-cs.1783 (PMC11157557; doi:10.7717/peerj-cs.1783)

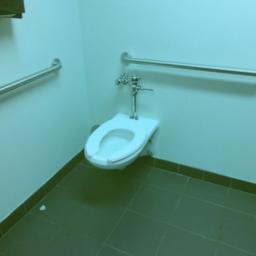

Supplement: Supplemental Information 2 — Experimental results of images on the test set. [file peerj-cs-10-1783-s002.zip › distorted/align_kv2_128_typeB.jpg]

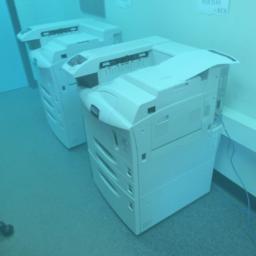

Supplement: Supplemental Information 2 — Experimental results of images on the test set. [file peerj-cs-10-1783-s002.zip › distorted/align_kv2_149_typeC.jpg]

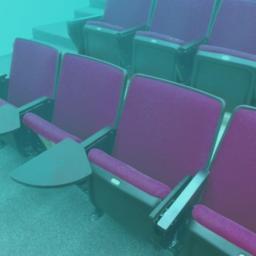

Supplement: Supplemental Information 2 — Experimental results of images on the test set. [file peerj-cs-10-1783-s002.zip › distorted/align_kv2_170_typeC.jpg]

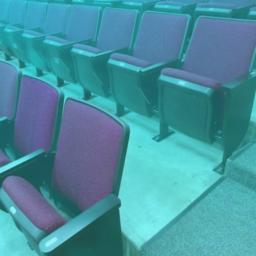

Supplement: Supplemental Information 2 — Experimental results of images on the test set. [file peerj-cs-10-1783-s002.zip › distorted/align_kv2_178_typeC.jpg]

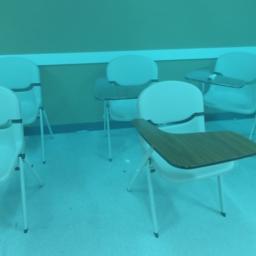

Supplement: Supplemental Information 2 — Experimental results of images on the test set. [file peerj-cs-10-1783-s002.zip › distorted/align_kv2_180_typeC.jpg]

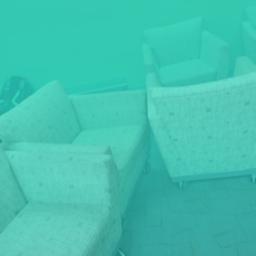

Supplement: Supplemental Information 2 — Experimental results of images on the test set. [file peerj-cs-10-1783-s002.zip › distorted/align_kv2_271_typeD.jpg]

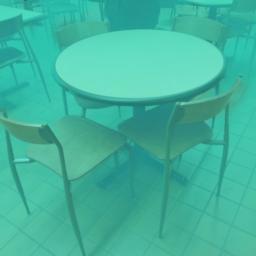

Supplement: Supplemental Information 2 — Experimental results of images on the test set. [file peerj-cs-10-1783-s002.zip › distorted/align_kv2_292_typeD.jpg]

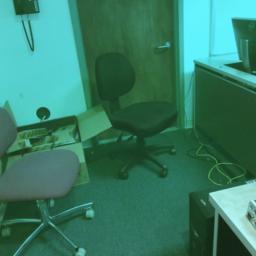

Supplement: Supplemental Information 2 — Experimental results of images on the test set. [file peerj-cs-10-1783-s002.zip › distorted/align_kv2_31_typeB.jpg]

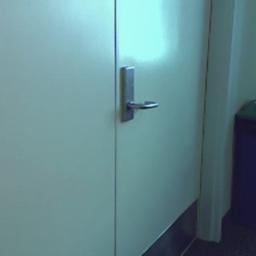

Supplement: Supplemental Information 2 — Experimental results of images on the test set. [file peerj-cs-10-1783-s002.zip › distorted/b3dodata_102_typeB.jpg]

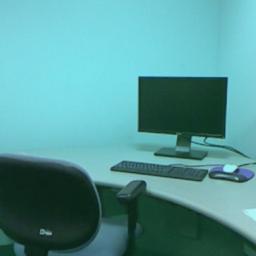

Supplement: Supplemental Information 2 — Experimental results of images on the test set. [file peerj-cs-10-1783-s002.zip › distorted/b3dodata_140_typeB.jpg]

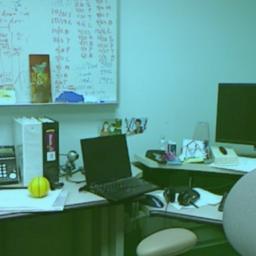

Supplement: Supplemental Information 2 — Experimental results of images on the test set. [file peerj-cs-10-1783-s002.zip › distorted/b3dodata_173_typeB.jpg]

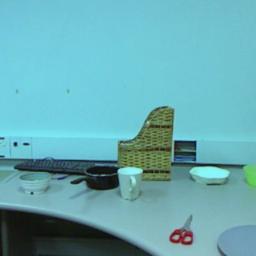

Supplement: Supplemental Information 2 — Experimental results of images on the test set. [file peerj-cs-10-1783-s002.zip › distorted/b3dodata_192_typeB.jpg]

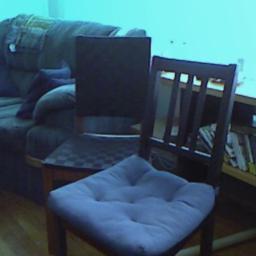

Supplement: Supplemental Information 2 — Experimental results of images on the test set. [file peerj-cs-10-1783-s002.zip › distorted/b3dodata_210_typeB.jpg]

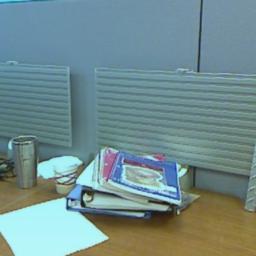

Supplement: Supplemental Information 2 — Experimental results of images on the test set. [file peerj-cs-10-1783-s002.zip › distorted/b3dodata_22_typeB.jpg]

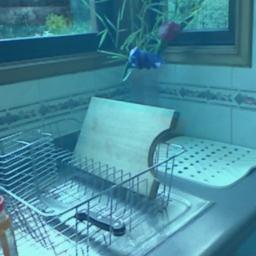

Supplement: Supplemental Information 2 — Experimental results of images on the test set. [file peerj-cs-10-1783-s002.zip › distorted/b3dodata_266_typeC.jpg]

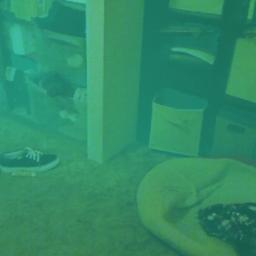

Supplement: Supplemental Information 2 — Experimental results of images on the test set. [file peerj-cs-10-1783-s002.zip › distorted/b3dodata_279_typeC.jpg]

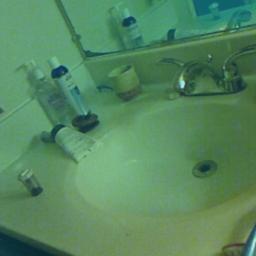

Supplement: Supplemental Information 2 — Experimental results of images on the test set. [file peerj-cs-10-1783-s002.zip › distorted/b3dodata_280_typeC.jpg]

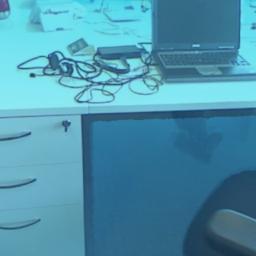

Supplement: Supplemental Information 2 — Experimental results of images on the test set. [file peerj-cs-10-1783-s002.zip › distorted/b3dodata_288_typeC.jpg]

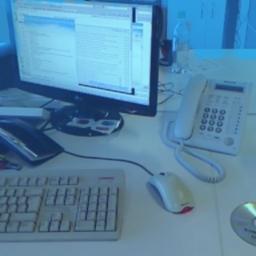

Supplement: Supplemental Information 2 — Experimental results of images on the test set. [file peerj-cs-10-1783-s002.zip › distorted/b3dodata_297_typeC.jpg]

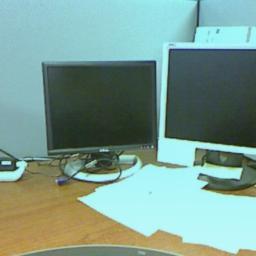

Supplement: Supplemental Information 2 — Experimental results of images on the test set. [file peerj-cs-10-1783-s002.zip › distorted/b3dodata_31_typeB.jpg]

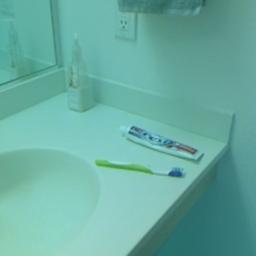

Supplement: Supplemental Information 2 — Experimental results of images on the test set. [file peerj-cs-10-1783-s002.zip › distorted/b3dodata_340_typeC.jpg]

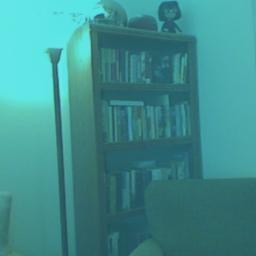

Supplement: Supplemental Information 2 — Experimental results of images on the test set. [file peerj-cs-10-1783-s002.zip › distorted/b3dodata_343_typeC.jpg]

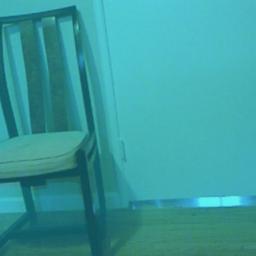

Supplement: Supplemental Information 2 — Experimental results of images on the test set. [file peerj-cs-10-1783-s002.zip › distorted/b3dodata_364_typeC.jpg]

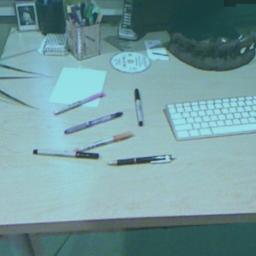

Supplement: Supplemental Information 2 — Experimental results of images on the test set. [file peerj-cs-10-1783-s002.zip › distorted/b3dodata_394_typeC.jpg]

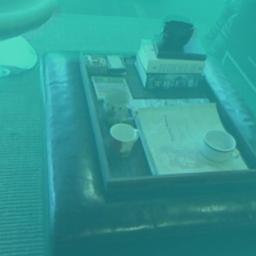

Supplement: Supplemental Information 2 — Experimental results of images on the test set. [file peerj-cs-10-1783-s002.zip › distorted/b3dodata_407_typeD.jpg]

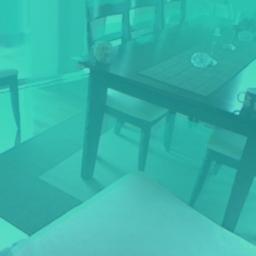

Supplement: Supplemental Information 2 — Experimental results of images on the test set. [file peerj-cs-10-1783-s002.zip › distorted/b3dodata_410_typeD.jpg]

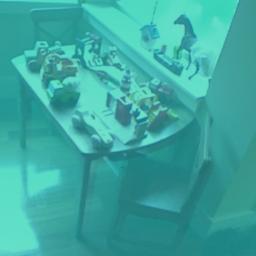

Supplement: Supplemental Information 2 — Experimental results of images on the test set. [file peerj-cs-10-1783-s002.zip › distorted/b3dodata_418_typeD.jpg]

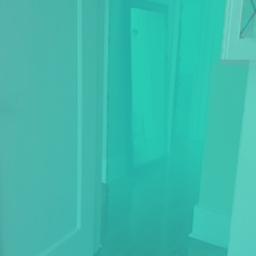

Supplement: Supplemental Information 2 — Experimental results of images on the test set. [file peerj-cs-10-1783-s002.zip › distorted/b3dodata_429_typeD.jpg]

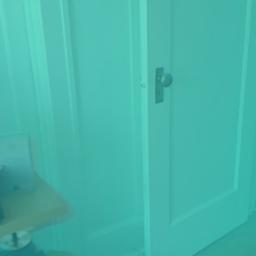

Supplement: Supplemental Information 2 — Experimental results of images on the test set. [file peerj-cs-10-1783-s002.zip › distorted/b3dodata_430_typeD.jpg]

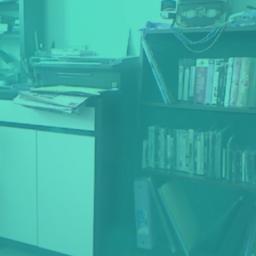

Supplement: Supplemental Information 2 — Experimental results of images on the test set. [file peerj-cs-10-1783-s002.zip › distorted/b3dodata_507_typeD.jpg]

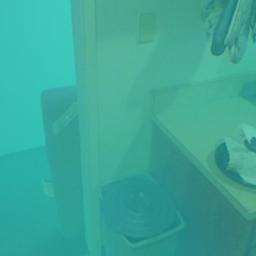

Supplement: Supplemental Information 2 — Experimental results of images on the test set. [file peerj-cs-10-1783-s002.zip › distorted/b3dodata_523_typeD.jpg]

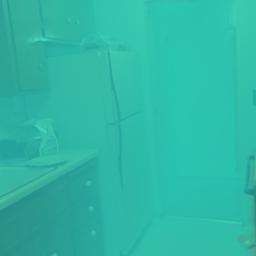

Supplement: Supplemental Information 2 — Experimental results of images on the test set. [file peerj-cs-10-1783-s002.zip › distorted/b3dodata_527_typeD.jpg]

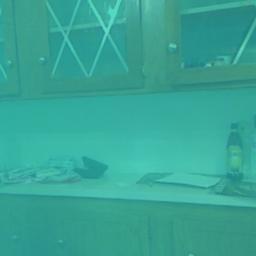

Supplement: Supplemental Information 2 — Experimental results of images on the test set. [file peerj-cs-10-1783-s002.zip › distorted/b3dodata_538_typeD.jpg]

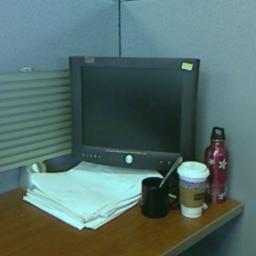

Supplement: Supplemental Information 2 — Experimental results of images on the test set. [file peerj-cs-10-1783-s002.zip › distorted/b3dodata_82_typeB.jpg]

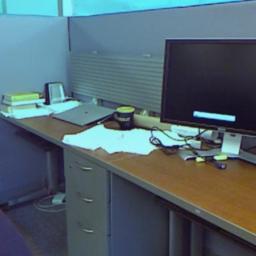

Supplement: Supplemental Information 2 — Experimental results of images on the test set. [file peerj-cs-10-1783-s002.zip › distorted/b3dodata_84_typeB.jpg]

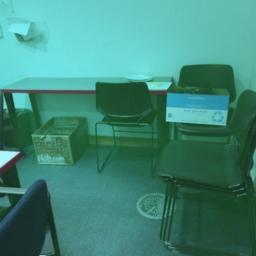

Supplement: Supplemental Information 2 — Experimental results of images on the test set. [file peerj-cs-10-1783-s002.zip › distorted/kinect2data_1002_typeB.jpg]

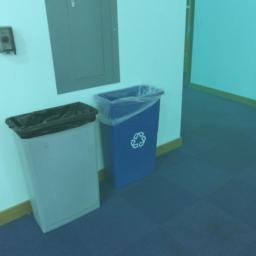

Supplement: Supplemental Information 2 — Experimental results of images on the test set. [file peerj-cs-10-1783-s002.zip › distorted/kinect2data_1006_typeB.jpg]

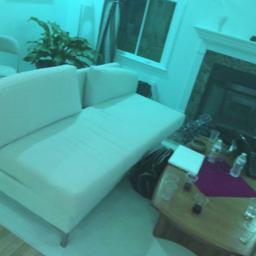

Supplement: Supplemental Information 2 — Experimental results of images on the test set. [file peerj-cs-10-1783-s002.zip › distorted/kinect2data_102_typeB.jpg]

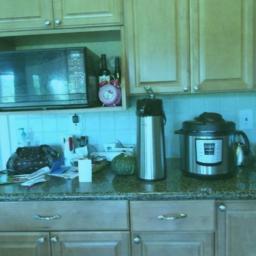

Supplement: Supplemental Information 2 — Experimental results of images on the test set. [file peerj-cs-10-1783-s002.zip › distorted/kinect2data_1035_typeB.jpg]

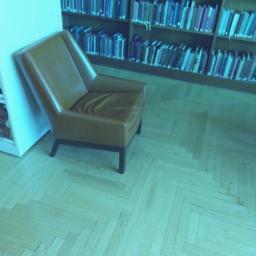

Supplement: Supplemental Information 2 — Experimental results of images on the test set. [file peerj-cs-10-1783-s002.zip › distorted/kinect2data_1069_typeB.jpg]

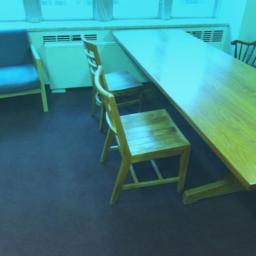

Supplement: Supplemental Information 2 — Experimental results of images on the test set. [file peerj-cs-10-1783-s002.zip › distorted/kinect2data_1108_typeB.jpg]

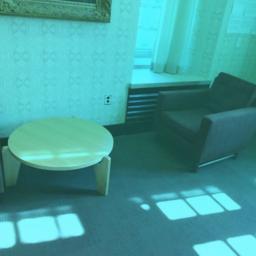

Supplement: Supplemental Information 2 — Experimental results of images on the test set. [file peerj-cs-10-1783-s002.zip › distorted/kinect2data_1132_typeB.jpg]

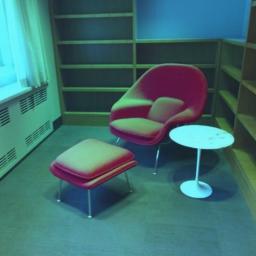

Supplement: Supplemental Information 2 — Experimental results of images on the test set. [file peerj-cs-10-1783-s002.zip › distorted/kinect2data_1137_typeB.jpg]

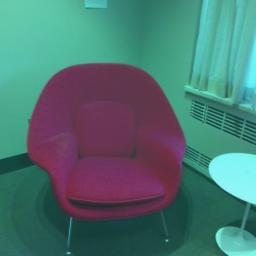

Supplement: Supplemental Information 2 — Experimental results of images on the test set. [file peerj-cs-10-1783-s002.zip › distorted/kinect2data_1138_typeB.jpg]

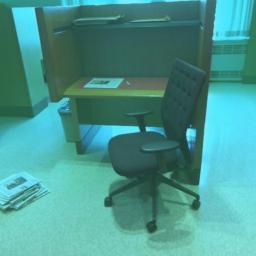

Supplement: Supplemental Information 2 — Experimental results of images on the test set. [file peerj-cs-10-1783-s002.zip › distorted/kinect2data_1144_typeB.jpg]

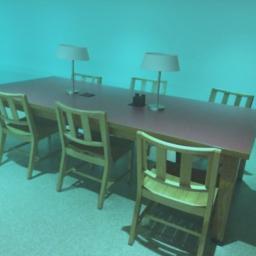

Supplement: Supplemental Information 2 — Experimental results of images on the test set. [file peerj-cs-10-1783-s002.zip › distorted/kinect2data_1153_typeB.jpg]

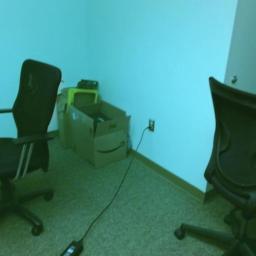

Supplement: Supplemental Information 2 — Experimental results of images on the test set. [file peerj-cs-10-1783-s002.zip › distorted/kinect2data_117_typeB.jpg]

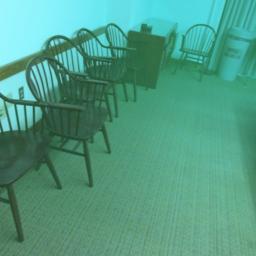

Supplement: Supplemental Information 2 — Experimental results of images on the test set. [file peerj-cs-10-1783-s002.zip › distorted/kinect2data_1242_typeB.jpg]

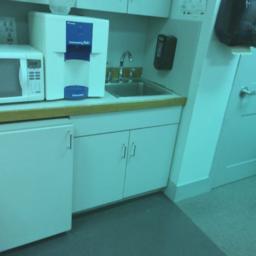

Supplement: Supplemental Information 2 — Experimental results of images on the test set. [file peerj-cs-10-1783-s002.zip › distorted/kinect2data_1281_typeB.jpg]

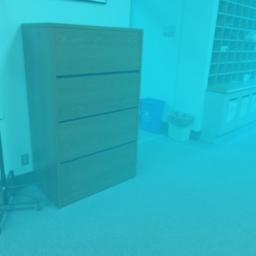

Supplement: Supplemental Information 2 — Experimental results of images on the test set. [file peerj-cs-10-1783-s002.zip › distorted/kinect2data_1287_typeC.jpg]

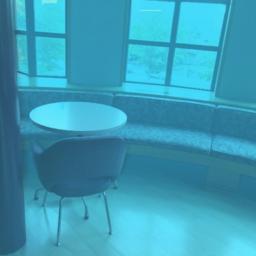

Supplement: Supplemental Information 2 — Experimental results of images on the test set. [file peerj-cs-10-1783-s002.zip › distorted/kinect2data_1294_typeC.jpg]

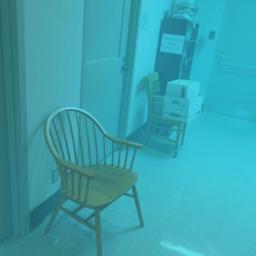

Supplement: Supplemental Information 2 — Experimental results of images on the test set. [file peerj-cs-10-1783-s002.zip › distorted/kinect2data_1315_typeC.jpg]

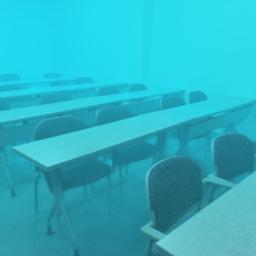

Supplement: Supplemental Information 2 — Experimental results of images on the test set. [file peerj-cs-10-1783-s002.zip › distorted/kinect2data_1324_typeC.jpg]

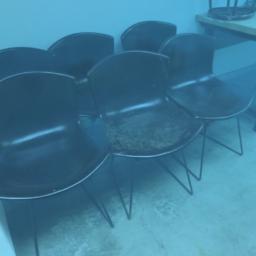

Supplement: Supplemental Information 2 — Experimental results of images on the test set. [file peerj-cs-10-1783-s002.zip › distorted/kinect2data_1451_typeC.jpg]

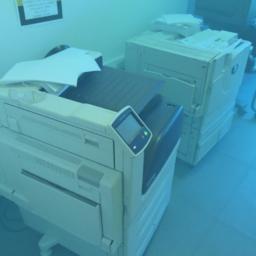

Supplement: Supplemental Information 2 — Experimental results of images on the test set. [file peerj-cs-10-1783-s002.zip › distorted/kinect2data_1462_typeC.jpg]

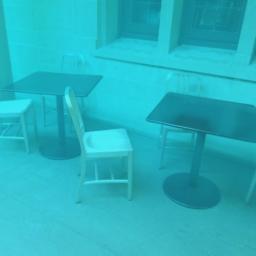

Supplement: Supplemental Information 2 — Experimental results of images on the test set. [file peerj-cs-10-1783-s002.zip › distorted/kinect2data_1470_typeC.jpg]

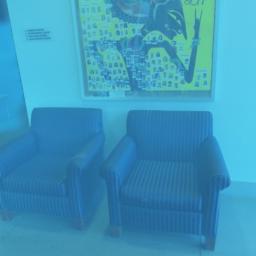

Supplement: Supplemental Information 2 — Experimental results of images on the test set. [file peerj-cs-10-1783-s002.zip › distorted/kinect2data_1491_typeC.jpg]

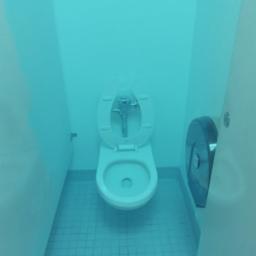

Supplement: Supplemental Information 2 — Experimental results of images on the test set. [file peerj-cs-10-1783-s002.zip › distorted/kinect2data_1508_typeC.jpg]

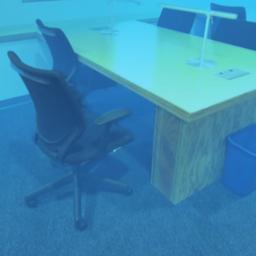

Supplement: Supplemental Information 2 — Experimental results of images on the test set. [file peerj-cs-10-1783-s002.zip › distorted/kinect2data_1534_typeC.jpg]

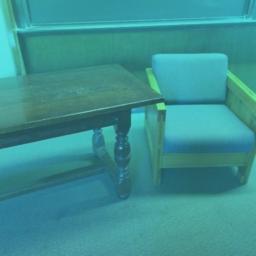

Supplement: Supplemental Information 2 — Experimental results of images on the test set. [file peerj-cs-10-1783-s002.zip › distorted/kinect2data_1545_typeC.jpg]

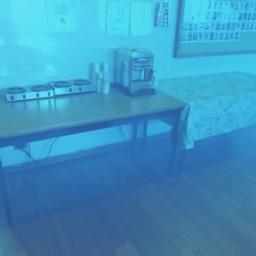

Supplement: Supplemental Information 2 — Experimental results of images on the test set. [file peerj-cs-10-1783-s002.zip › distorted/kinect2data_1558_typeC.jpg]

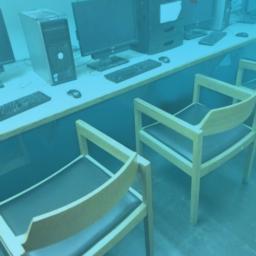

Supplement: Supplemental Information 2 — Experimental results of images on the test set. [file peerj-cs-10-1783-s002.zip › distorted/kinect2data_1566_typeC.jpg]

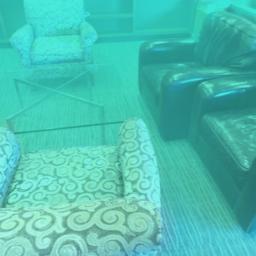

Supplement: Supplemental Information 2 — Experimental results of images on the test set. [file peerj-cs-10-1783-s002.zip › distorted/kinect2data_1575_typeC.jpg]

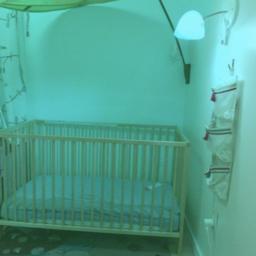

Supplement: Supplemental Information 2 — Experimental results of images on the test set. [file peerj-cs-10-1783-s002.zip › distorted/kinect2data_168_typeB.jpg]

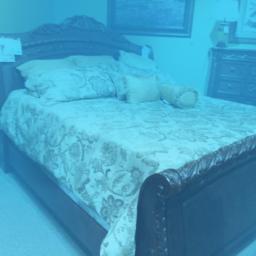

Supplement: Supplemental Information 2 — Experimental results of images on the test set. [file peerj-cs-10-1783-s002.zip › distorted/kinect2data_1714_typeC.jpg]

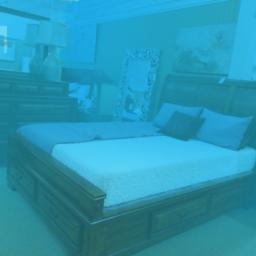

Supplement: Supplemental Information 2 — Experimental results of images on the test set. [file peerj-cs-10-1783-s002.zip › distorted/kinect2data_1782_typeC.jpg]

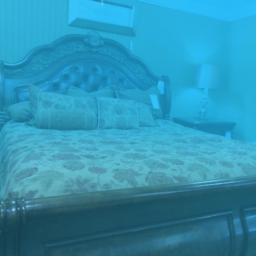

Supplement: Supplemental Information 2 — Experimental results of images on the test set. [file peerj-cs-10-1783-s002.zip › distorted/kinect2data_1816_typeC.jpg]

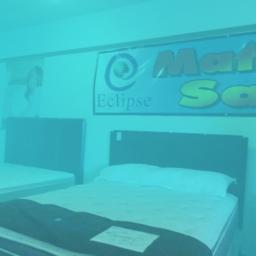

Supplement: Supplemental Information 2 — Experimental results of images on the test set. [file peerj-cs-10-1783-s002.zip › distorted/kinect2data_1894_typeC.jpg]

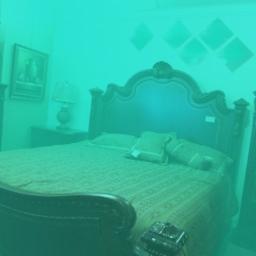

Supplement: Supplemental Information 2 — Experimental results of images on the test set. [file peerj-cs-10-1783-s002.zip › distorted/kinect2data_1931_typeC.jpg]

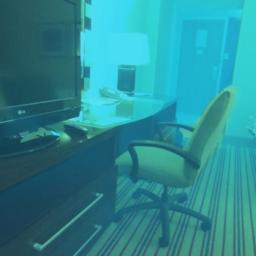

Supplement: Supplemental Information 2 — Experimental results of images on the test set. [file peerj-cs-10-1783-s002.zip › distorted/kinect2data_1941_typeC.jpg]

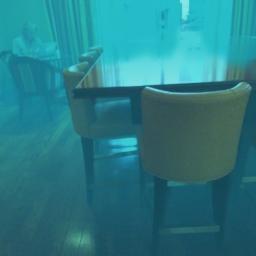

Supplement: Supplemental Information 2 — Experimental results of images on the test set. [file peerj-cs-10-1783-s002.zip › distorted/kinect2data_1948_typeC.jpg]

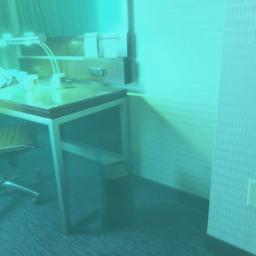

Supplement: Supplemental Information 2 — Experimental results of images on the test set. [file peerj-cs-10-1783-s002.zip › distorted/kinect2data_1988_typeC.jpg]

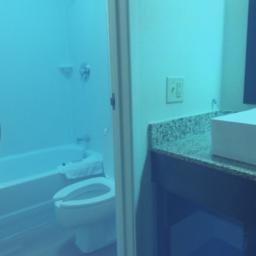

Supplement: Supplemental Information 2 — Experimental results of images on the test set. [file peerj-cs-10-1783-s002.zip › distorted/kinect2data_2058_typeC.jpg]

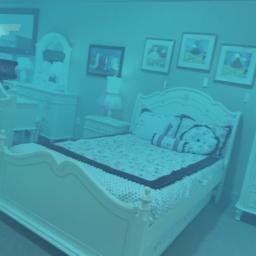

Supplement: Supplemental Information 2 — Experimental results of images on the test set. [file peerj-cs-10-1783-s002.zip › distorted/kinect2data_2116_typeC.jpg]

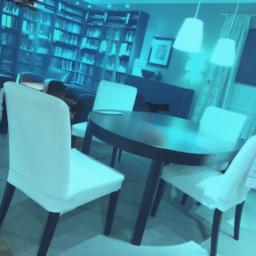

Supplement: Supplemental Information 2 — Experimental results of images on the test set. [file peerj-cs-10-1783-s002.zip › distorted/kinect2data_220_typeB.jpg]

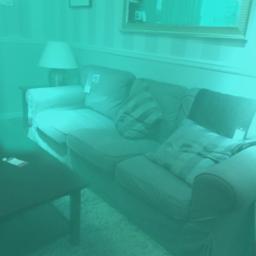

Supplement: Supplemental Information 2 — Experimental results of images on the test set. [file peerj-cs-10-1783-s002.zip › distorted/kinect2data_2222_typeC.jpg]

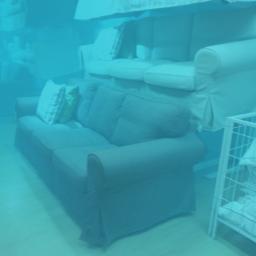

Supplement: Supplemental Information 2 — Experimental results of images on the test set. [file peerj-cs-10-1783-s002.zip › distorted/kinect2data_2229_typeC.jpg]

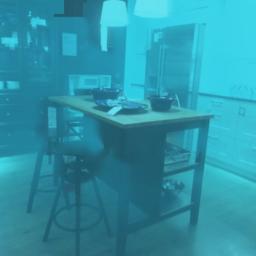

Supplement: Supplemental Information 2 — Experimental results of images on the test set. [file peerj-cs-10-1783-s002.zip › distorted/kinect2data_2252_typeC.jpg]

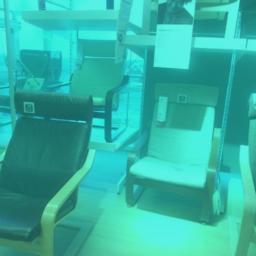

Supplement: Supplemental Information 2 — Experimental results of images on the test set. [file peerj-cs-10-1783-s002.zip › distorted/kinect2data_2253_typeC.jpg]

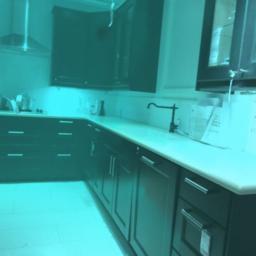

Supplement: Supplemental Information 2 — Experimental results of images on the test set. [file peerj-cs-10-1783-s002.zip › distorted/kinect2data_225_typeB.jpg]

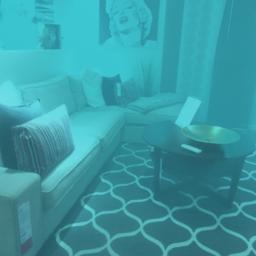

Supplement: Supplemental Information 2 — Experimental results of images on the test set. [file peerj-cs-10-1783-s002.zip › distorted/kinect2data_2260_typeC.jpg]

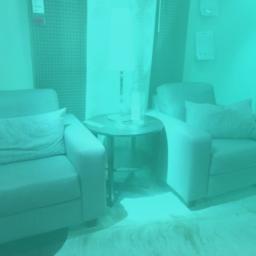

Supplement: Supplemental Information 2 — Experimental results of images on the test set. [file peerj-cs-10-1783-s002.zip › distorted/kinect2data_2262_typeC.jpg]

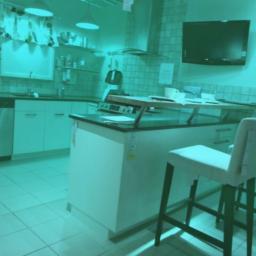

Supplement: Supplemental Information 2 — Experimental results of images on the test set. [file peerj-cs-10-1783-s002.zip › distorted/kinect2data_226_typeB.jpg]

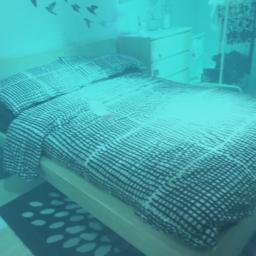

Supplement: Supplemental Information 2 — Experimental results of images on the test set. [file peerj-cs-10-1783-s002.zip › distorted/kinect2data_2302_typeC.jpg]

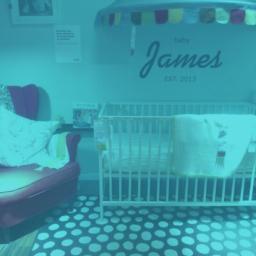

Supplement: Supplemental Information 2 — Experimental results of images on the test set. [file peerj-cs-10-1783-s002.zip › distorted/kinect2data_2313_typeC.jpg]

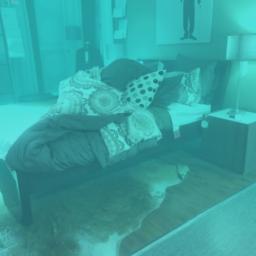

Supplement: Supplemental Information 2 — Experimental results of images on the test set. [file peerj-cs-10-1783-s002.zip › distorted/kinect2data_2314_typeC.jpg]

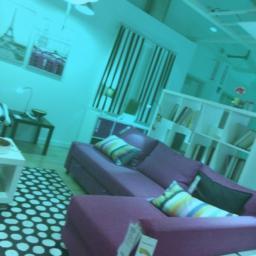

Supplement: Supplemental Information 2 — Experimental results of images on the test set. [file peerj-cs-10-1783-s002.zip › distorted/kinect2data_231_typeB.jpg]

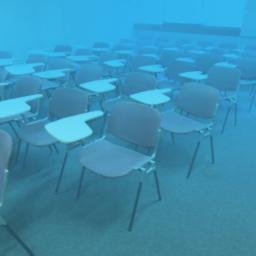

Supplement: Supplemental Information 2 — Experimental results of images on the test set. [file peerj-cs-10-1783-s002.zip › distorted/kinect2data_2410_typeC.jpg]

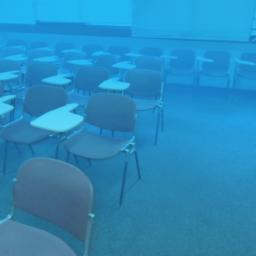

Supplement: Supplemental Information 2 — Experimental results of images on the test set. [file peerj-cs-10-1783-s002.zip › distorted/kinect2data_2414_typeC.jpg]

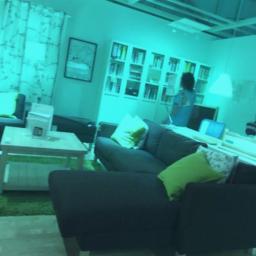

Supplement: Supplemental Information 2 — Experimental results of images on the test set. [file peerj-cs-10-1783-s002.zip › distorted/kinect2data_241_typeB.jpg]

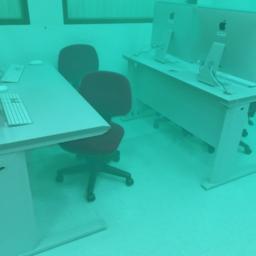

Supplement: Supplemental Information 2 — Experimental results of images on the test set. [file peerj-cs-10-1783-s002.zip › distorted/kinect2data_2442_typeC.jpg]

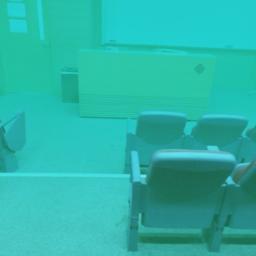

Supplement: Supplemental Information 2 — Experimental results of images on the test set. [file peerj-cs-10-1783-s002.zip › distorted/kinect2data_2495_typeC.jpg]

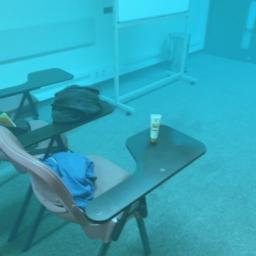

Supplement: Supplemental Information 2 — Experimental results of images on the test set. [file peerj-cs-10-1783-s002.zip › distorted/kinect2data_2522_typeC.jpg]

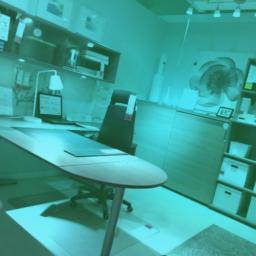

Supplement: Supplemental Information 2 — Experimental results of images on the test set. [file peerj-cs-10-1783-s002.zip › distorted/kinect2data_252_typeB.jpg]

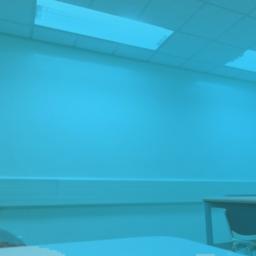

Supplement: Supplemental Information 2 — Experimental results of images on the test set. [file peerj-cs-10-1783-s002.zip › distorted/kinect2data_2531_typeC.jpg]

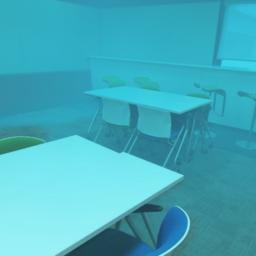

Supplement: Supplemental Information 2 — Experimental results of images on the test set. [file peerj-cs-10-1783-s002.zip › distorted/kinect2data_2545_typeC.jpg]

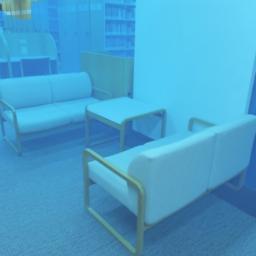

Supplement: Supplemental Information 2 — Experimental results of images on the test set. [file peerj-cs-10-1783-s002.zip › distorted/kinect2data_2570_typeC.jpg]

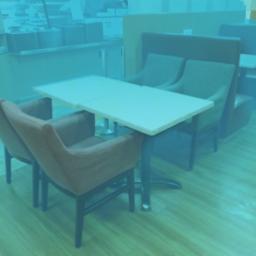

Supplement: Supplemental Information 2 — Experimental results of images on the test set. [file peerj-cs-10-1783-s002.zip › distorted/kinect2data_2583_typeC.jpg]

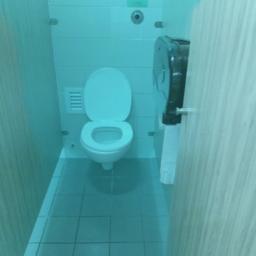

Supplement: Supplemental Information 2 — Experimental results of images on the test set. [file peerj-cs-10-1783-s002.zip › distorted/kinect2data_2588_typeC.jpg]

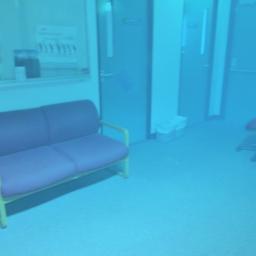

Supplement: Supplemental Information 2 — Experimental results of images on the test set. [file peerj-cs-10-1783-s002.zip › distorted/kinect2data_2596_typeC.jpg]
